# The impact of reclassification of C3 predominant glomerulopathies on diagnostic accuracy, outcome and prognosis in patients with C3 glomerulonephritis

**DOI:** 10.1186/s12882-020-01923-5

**Published:** 2020-07-11

**Authors:** P. Puri, G. D. Walters, M. N. Fadia, M. Konia, K. A. Gibson, S. H. Jiang

**Affiliations:** 1 Department of Renal Medicine, The Canberra Hospital, Canberra, ACT Australia; 2 Department of Immunology and Infectious Disease, John Curtin School of Medical Research, Canberra, ACT Australia; 3 Centre for Personalised Immunology, NHMRC Centre for Research Excellence, Canberra, Australia; 4 Australian National University, Canberra, ACT 2601 Australia; 5 Department of Anatomical and histopathology, The Canberra Hospital, Canberra, ACT Australia

**Keywords:** C3 glomerulonephritis, Membranoproliferative glomerulonephritis, Electron microscopy

## Abstract

**Background:**

C3 glomerulonephritis is a recently described entity with heterogeneous histopathological features. This study was conducted to assess the effect of reclassification of C3 glomerulopathies on renal outcomes, mortality, and response to therapy.

**Methods:**

We undertook a retrospective analysis of 857 renal biopsies collected at The Canberra Hospital. Samples with predominant C3 staining were reviewed by a renal histopathologist. Of 31 biopsies with predominant C3 staining, 10 fulfilled histological criteria for C3 glomerulonephritis, while the remaining 21 cases were used as C3 Controls.

**Results:**

Aside from a higher incidence of C3 glomerulonephritis in Torres Strait islanders (40% vs 5% C3 Controls, *p* = 0.04), presentation demographics were similar between the two groups. Median creatinine at diagnosis was higher in patients with C3 glomerulonephritis (253 umol/L IQR 103–333 vs 127 umol/L C3 Controls, IQR 105–182, *p* = 0.01).

Prior to reclassification, a majority of C3 glomerulonephritis cases were diagnosed as membranoproliferative glomerulonephritis (60% vs 5% (C3 Controls) *p* < 0.01). Electron microscopy demonstrated all C3 glomerulonephritis patients had C3 deposition (100% vs 38% *p* = 0.02), these deposits were amorphous in nature (50% vs 5% respectively *p* = 0.007).

C3 glomerulonephritis patients had shorter median follow-up (405 days IQR 203–1197 vs 1822 days respectively, IQR 1243–3948, *p* = 0.02). Mortality was higher in C3 glomerulonephritis patients (30% vs 14% in C3 Controls (log rank p = 0.02)).

**Conclusion:**

We have devised a diagnostic and treatment algorithm based on the results of literature review and our current study. Further prospective assessment is required to review diagnostic and treatment outcomes for this disease in Australian centres.

## Background

Membranoproliferative glomerulonephritis (MPGN) is an uncommon cause of immune-mediated kidney disease [1]. MPGN has been typically defined by the histopathological lesions of mesangial hypercellularity, endocapillary proliferation, and duplication of the basement membrane [1–3]. It was subclassified by the localisation of deposits on electron microscopy (EM) into type I, type II, and type III MPGN [2, 4]. However, advances in the understanding of immunoglobulin and complement-mediated glomerular disease have led to the proposed reclassification of MPGN, utilising histological findings in addition to evidence of the underlying pathophysiological processes [1, 3]. These include: immune complex-mediated disease (IC), complement dysregulation and overactivation, or neither complement nor IC-mediated endothelial injury [2, 4]. IC-medicated MPGN is the most common mechanism for disease and is typically associated with infection, monoclonal gammopathies or autoimmune disease [3–6]. MPGN arising in the absence of IC or complement deposition occurs in the context of thrombotic microangiopathy, seen in haemolytic uremic syndrome and malignant hypertension [4].

MPGN due to dysregulation of the alternate complement pathway, now termed C3 glomerulopathy (C3G), is less common than the IC-mediated type, with a reported incidence between 1 and 3 cases per million [4–6]. Its defining characteristic is predominant C3 deposition within the mesangium or capillary wall, detected by immunofluorescence (IF) staining which is two orders of magnitude greater than any other IF reactant [4]. C3G can be further divided into dense deposit diseases (DDD) and C3 glomerulonephritis (C3GN) [1] by electron microscopic findings. DDD (formerly, type II MPGN) is defined by electron-dense intramembranous deposits along the glomerular basement membrane (GBM), Bowman’s capsule and tubular basement membrane [1, 4, 5] whereas C3GN (formerly, either type I or III MPGN) is defined by amorphous C3 deposition within the capillary wall and mesangium [1–3, 6].

C3GN shares significant morphological overlap with resolving post-infectious GN (PIGN), and thus distinguishing these can be challenging [7, 8]. Like C3GN, atypical PIGN may lack immunoglobulin deposition and be C3 positive in a similar pattern [5, 9]. Given the histopathological similarities, these conditions are distinguished by clinical features such as history of recent infection, anti-streptolysin titres (ASOT), serial complement levels, complement regulator protein analysis and investigation for underlying haematological or connective tissue diseases [7–9].

Cohort studies in the United States, Europe and Asia have led to understanding of disease progression and treatment outcomes of C3GN [7, 9–12]. Currently, there is no data on Australian patients with C3GN or disease progression. Here we explore whether reclassification of historical renal biopsy samples might be linked to outcomes in kidney function in an Australian tertiary centre. This study presents retrospective data on biopsies that have been reassessed based upon the reclassification of C3G. We assess the effect of clinical plus histological features and whether reclassification improves the association with disease outcome or progression [8, 12–15].

## Methods

The study was approved by the Australian Capital territory’s (ACT) Health Human Research Ethics Committee. Renal biopsy reports at a single centre from 2000 to 2018 were retrospectively reviewed to identify biopsies with predominant C3 staining that may be consistent with C3 glomerulopathy [1–3, 14]. C3 glomerulopathy was defined as C3 positivity two orders of magnitude above any other immunoreactant on immunofluorescence [1–3].

**Fig. 1:**
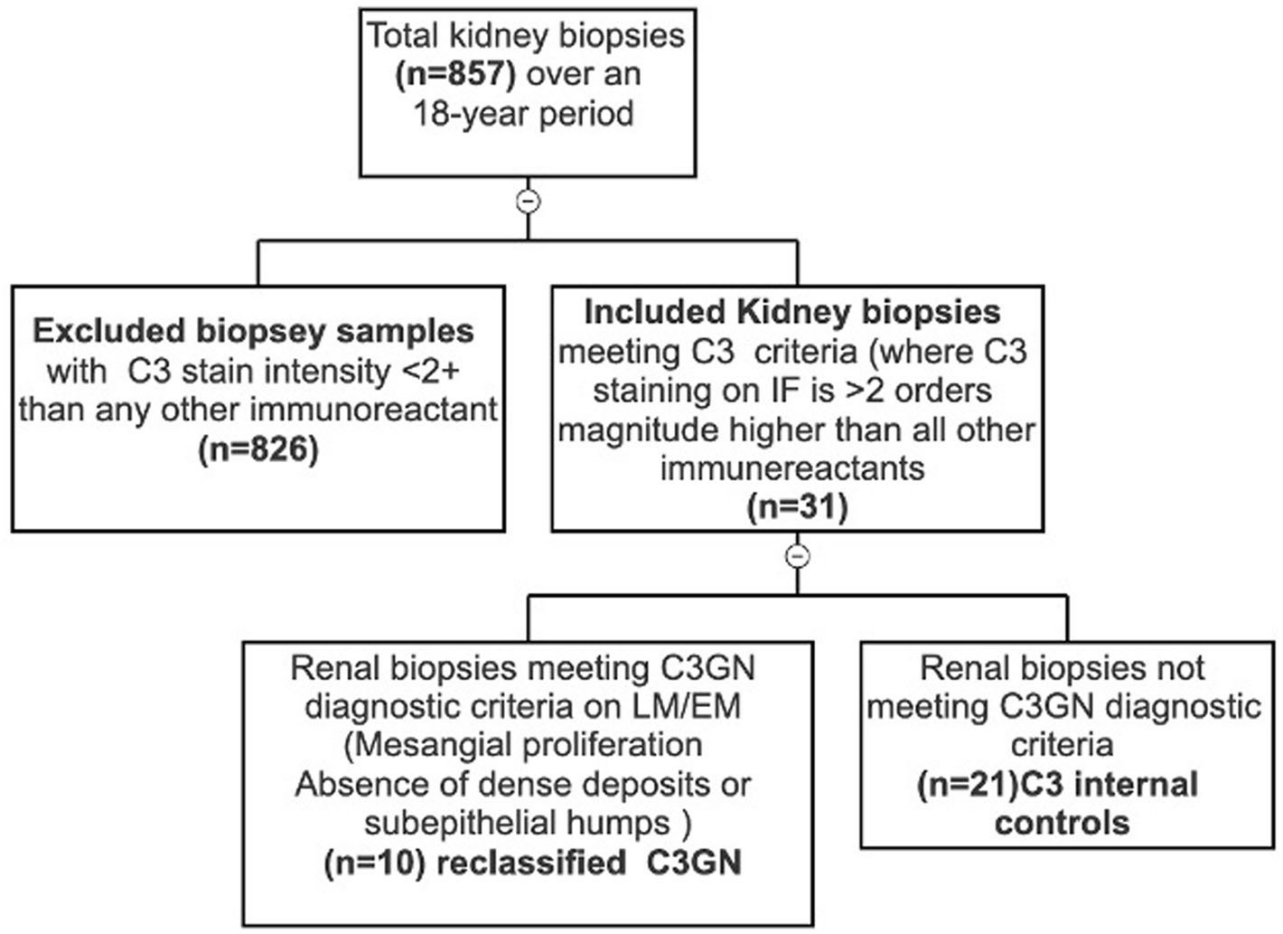
section criteria of renal biopsies assessed for reclassification. C3 predominant samples were considered C3 + > 2 of magnitudes higher than any other immunereactant on IF

**Fig. 2:**
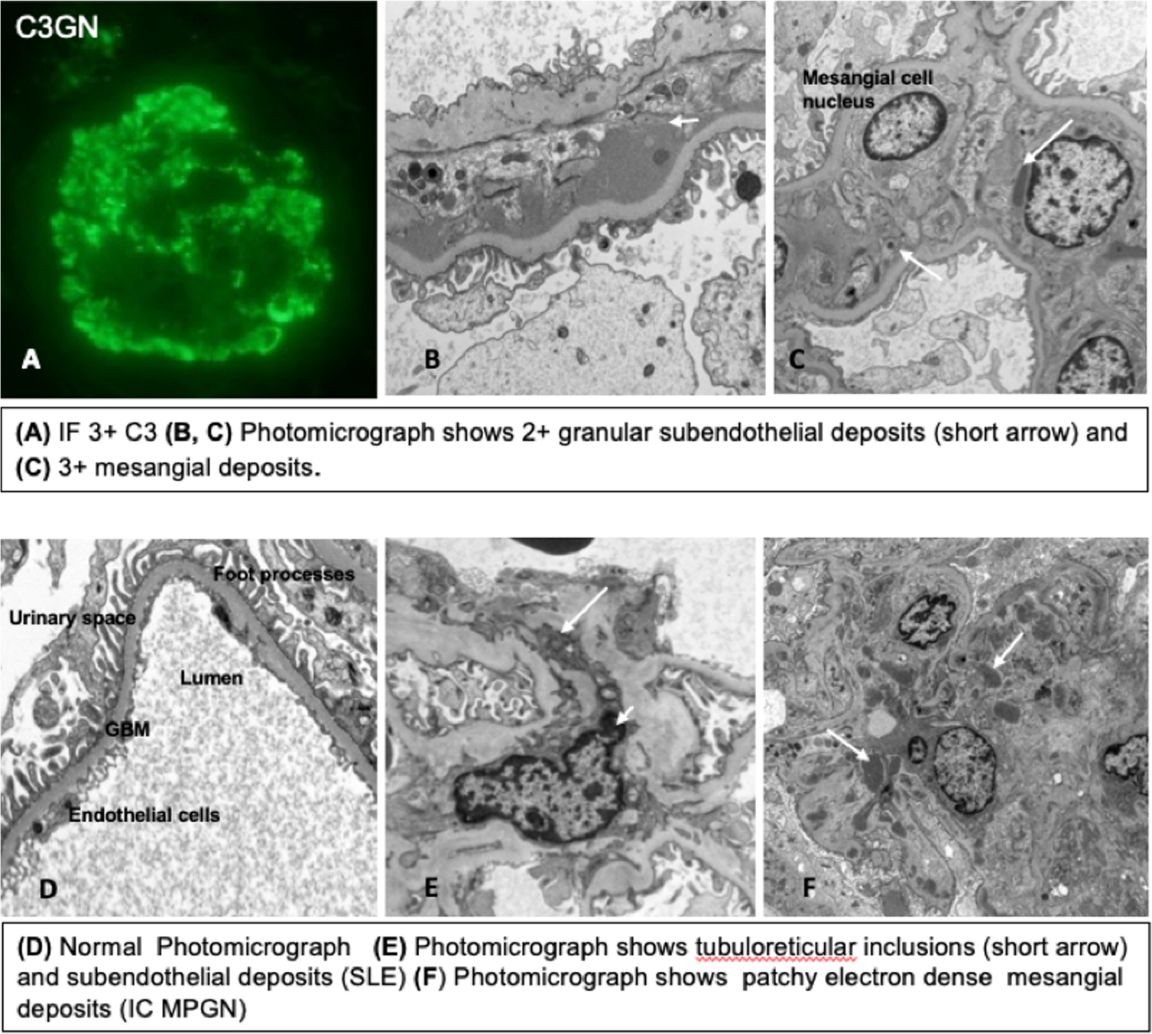
Histological features of samples used A-C is example of one C3GN patient D-F normal healthy Control, and two C3 Controls

Biopsies with C3 predominance were re-processed and assessed by IF, light microscopy (LM) and EM by a single nephropathologist (MF) and two nephrologists (SJ, GW). Immunoreactants measured by IF were scored by increasing intensity of fluorescence (0–3+). EM was used to assess the morphology of deposition and to determine the presence of dense intramembranous deposits or multiple subepithelial humps. Mesangial or endocapillary proliferation in the presence of predominant C3 deposition, in the absence of dense deposits or subepithelial humps were considered to be C3GN. Samples that met IF (C3 predominance) but not EM criteria were subsequently used as internal histological Controls (C3 Controls) (Figs. 1 and 2). A total of 10 biopsies were reclassified as C3GN while 21 meeting IF criteria only were considered C3 Controls.

Treatment outcomes assessed included remission (defined as urine protein/creatinine ratio (uPCR) < 0.5 g/day) and partial remission (defined as uPCR < 3.5 g/day plus a 50% reduction in creatinine), chronic haemodialysis (HD) for > 3 months, transplant, or all-cause mortality. Acute Kidney injury (AKI) was defined in concordance with Kidney Diseases Improving Global Outcomes (KDIGO) [16] definitions as an increase in serum creatinine by > 26.5 umol/l within 48 h, or an increase in serum creatinine to 1.5 times baseline, which is known or presumed to have occurred within the prior 7 days.

### Statistical analysis

Continuous variables were expressed as a median score and compared using a Mann-Whitney U test. Categorical variables were presented as percentage of total group and were compared using Fisher’s exact test. The Kaplan–Meier method was used to determine survival and log-rank testing was performed to compare groups. A *P* value of < 0.05 was considered statistically significant. Data were analysed using IBM SPSS Version 23, 2015.

## Results

### Reclassification

Eight hundred fifty-seven renal biopsy reports were reviewed, and 31 biopsies identified with predominant C3 staining on IF (Figs. 1 and 2). The clinical presentation, treatment, and outcomes were assessed for each of the 31 patients. Ten of 31 biopsies examined were consistent with C3GN, whilst the remaining 21 samples were consistent with C3 mediated disease but not consistent with C3GN (Figs. 1 and 2).

**Fig. 3:**
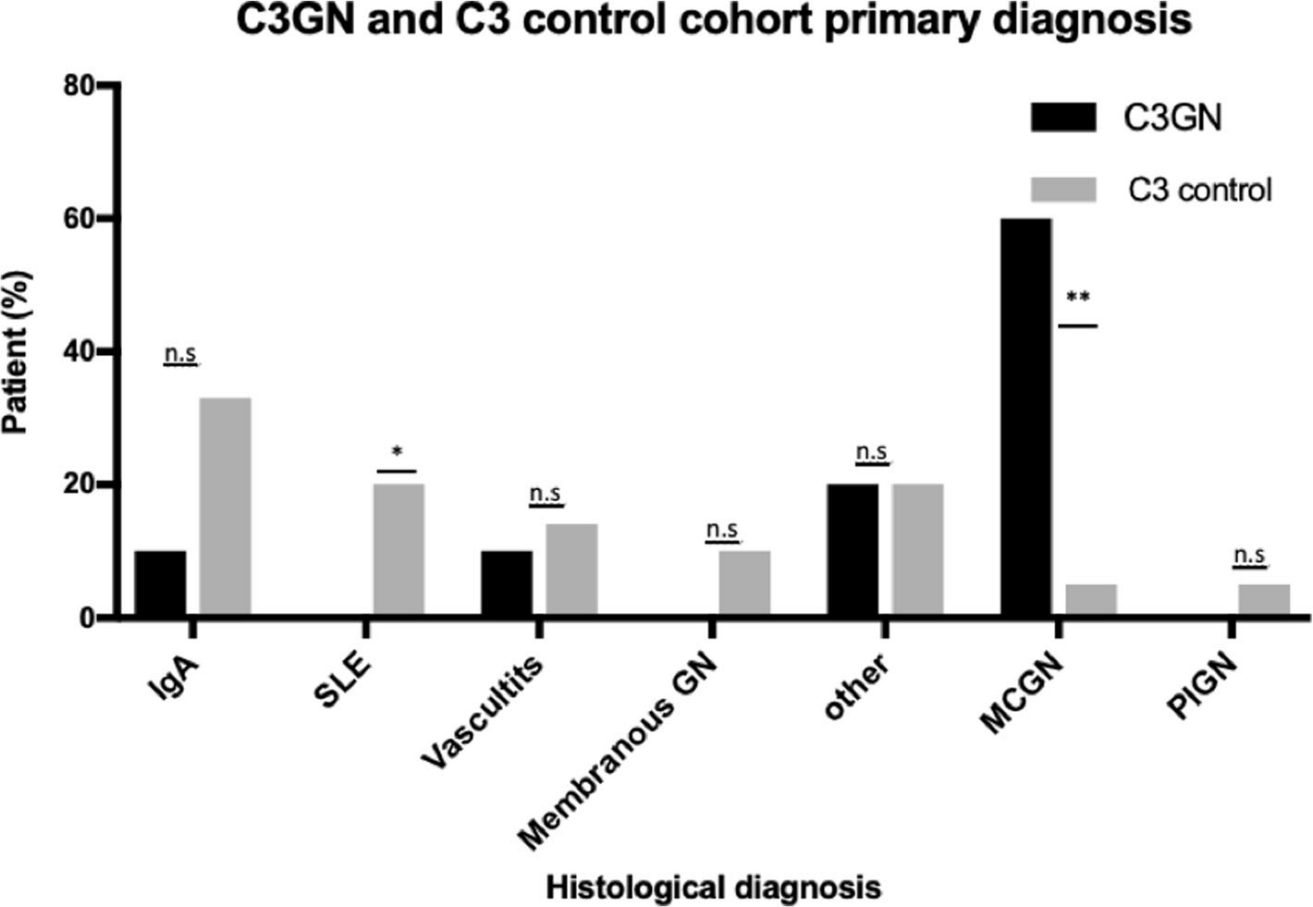
C3GN and C3 Control cohort’s original diagnosis at biopsy prior to reclassification. *P* value < 0.05 = *, p < 0.01 = **, *p* < 0.001 = *** *P* > 0.5 = n.s

We evaluated the original histopathological diagnosis from histology reports. C3GN cases were originally reported as MPGN in most patients compared to C3 Controls (60% vs 5%, *p* < 0.01). There were no cases of Lupus nephritis (LN) reported in the C3GN group, however 20% of C3 Controls were reported as having LN (*p* = 0.05) (Fig. 3). C3 Controls had a broader spectrum of diagnoses at the time of biopsy compared to C3GN patients (Fig. 3).

### Baseline data

We assessed the characteristics of patients reclassified by histopathology. The mean age at diagnosis was similar between C3GN and C3 Control groups (57 vs 55 years, *p* = 0.9). There was no significant gender difference between C3GN and C3 Control groups (Table 1). In relation to self-reported ethnicity we observed an increased incidence of C3GN in patients of Torres Strait islander descent (40% vs 5%, *p* = 0.04), however Caucasians comprised a majority of patients in both groups 60% vs 85%, *p* = 0.08) (Table 1).

**Table 1:** Baseline characteristics of C3GN and C3 Control patients

Category	C3GN (***n*** = 10)	C3 Controls (***n*** = 21)	***p*** value
**Gender (% Male)**	50 (*n* = 5)	33(*n* = 7)	0.4
**Age at diagnosis (years)**	57 (41–61)	55 (35–65)	0.9
**SBP (mmHg)**	140 (122–160)	145 (128–154)	0.9
**DBP (mmHg)**	83 (68–89)	80 (70–87)	0.7
**Caucasian (%)**	60 (*n* = 6)	85 (*n* = 18)	0.08
**Asian (%)**	0	10 (n = 2)	0.2
**Aboriginal (%)**	0	0	–
**Torres Strait Islander (%)**	10 (n = 1)	5 (*n* = 1)	0.04
**Hispanic (%)**	0	5	0.3
**Black (%)**	0	0	–
**AKI (%)**	60 (n = 6)	57 (*n* = 12)	0.9
**AKI needing acute HD (%)**	30 (*n* = 30)	14 (*n* = 3)	0.3
**Nephrotic syndrome (%)**	30 (n = 30)	10 (n = 2)	0.3
**Haematuria (%)**	100 (n = 10)	80 (*n* = 17)	0.3
**uPCR at diagnosis (mg/mmol)**	300 (106–947)	154 (55–573)	0.7
**Creatinine at diagnosis (umol/L)**	253 (103–333)	127 (105–182)	0.01
**Complement levels (C3)**	0.91 (0.56–1.04)	1.15 (0.93–1.39)	0.4
**Low Complement levels (%)**	20	19	1
**ANA titre (dilution)**	80 (80–329)	80 (0–200)	0.6
**ANCA (% positive)**	0	20	0.2
**C3Nef/CFH/CFb antibody (%)**	0 (n = 0/2)	0 not tested	–
**Significant light chain ratio (% positive)**	0 (n = 0/4)	0 (*n* = 0/7)	–
**ASOT titre (+) > 200 IU (%)**	0 (n = 0/2)	50 (n = 1/2)	0.2

All data reported in median plus IQR unless otherwise indicated

### Features on presentation

There was no difference in median blood pressure or age at diagnosis between the two groups. In terms of disease severity, 30% of C3GN patients presented with nephrotic syndrome compared to 10% of C3 Controls (*p* = 0.3) (Table 1). Haematuria was nearly equally prevalent in C3GN patients and C3 Controls (100% vs 80%, p = 0.3), proteinuria appeared to be increased in C3GN group compared to Controls, however, was not statistically significant (uPCR 300 mg/mmol IQR 106–947 vs 154 mg/mmol IQR 55–573, *p* = 0.7). Serum creatinine at presentation was higher in the C3GN group (253 umol/L, IQR:103–333 vs 127 umol/L, IQR:105–182, *p* = 0.01) which is not consistent with other reports [7, 8]. Acute kidney injury at presentation was found in 60% C3GN patients and 57% of C3 Controls (*p* = 0.9). A requirement for dialysis occurred in 30% of C3GN patients at diagnoses as compared to 14% of C3 Controls (*p* = 0.3).

A comparison of serological results showed no statistical difference in baseline ANA (median 1:80) and C3 titre (0.91, IQR = 0.56–1.04 vs 1.15, IQR = 0.93–1.39, *p* = 0.4) respectively between the two groups (Table 1). Anti-streptolysin titres (ASOT) were negative in two C3GN patients, but positive at a low titre (681 IU) for one of two Control patients tested (*p* = 0.2). Myeloma screening was completed in 40% C3GN patients, and 33% Controls with no detectable paraprotein or haematological malignancies at the time of diagnosis (Table 1).

### Histological findings

We next examined the histopathologic findings in biopsy samples. LM features of the 10 patients reclassified into C3GN revealed 60% of C3GN cohort had an MPGN pattern of injury compared to 5% of C3 Controls (*p* < 0.01, Table 2). On IF, significantly more C3GN patients had C3 deposition alone versus the C3 Control group (80% vs 24% *p* = 0.05) (Table 2). We observed a significantly greater of portion amorphous deposits on EM seen in the C3GN group compared to the C3 Control group (50% vs 5%, *p* = 0.007) (Table 2).

**Table 2:** Features of histological scoring and features of C3GN and C3 Control biopsy samples

Histology features	C3GN patients (***n*** = 10)	C3 Control (***n*** = 21)	***p*** value
**Light Microscopy patterns**			
Normal (%)	10 (n = 1)	14 (n = 3)	1
MPGN (%)	60 (n = 6)	5 (n = 1)	< 0.01
Mesangial proliferation (%)	30 (n = 3)	57 (n = 12)	0.3
Endocapillary proliferation (%)	40 (*n* = 4)	14 (n = 3)	0.2
Leucocyte infiltration (%)	30 (n = 3)	19 (*n* = 4)	0.7
Cellular or fibrocellular crescents (%)	30 (n = 3)	38 (n = 7)	1
**Immunofluorescence**			
C3 alone **(%)**	80 (*n* = 8)	24 (n = 5)	0.05
C3 dominant (with trace or 1+ Ig) **(%)**	20 (n = 2)	38 (n = 7)	0.4
C3 dominant (with trace > 2+ Ig) **(%)**	0	38 (n = 7)	0.03
**Electron microscopy findings**			
No deposits **(%)**	0	38 (n = 7)	0.02
Any deposits **(%)**	100 (n = 10)	62 (*n* = 13)	0.5
Mesangial **(%)**	40 (n = 4)	19 (n = 4)	0.7
Subendothelial **(%)**	20 (n = 2)	24 (n = 5)	0.6
Subepithelial **(%)**	10 (n = 1)	5 (*n* = 1)	1.00
All 3 locations **(%)**	30 (n = 3)	14 (*n* = 3)	0.4
**Deposit features**			
Amorphous **(%)**	50 (n = 5)	5 (n = 1)	0.007
Dense **(%)**	10 (n = 1)	14 (n = 3)	0.6
Patchy **(%)**	30 (n = 3)	52 (*n* = 11)	0.2
Subendothelial humps **(%)**	10 (n = 1)	30 (n = 6)	0.4

### Treatment outcomes

Patients reclassified as C3GN had shorter median follow-up compared to C3 Controls (405 days IQR 203–1197 vs 1822 days IQR 1243–3948, *p* = 0.01) (Table 3). This could be attributed to the fact there were 30% patients lost to follow up from the C3GN group. Creatinine doubled in 10% of C3GN vs 19% of C3 Controls (*p* = 0.2), time to doubling of serum creatinine was times longer for C3 Control patients though this was not statistically significant (350 vs 1107 days *p* = 0.3, respectively). Immunosuppression was administered in 40% of the C3GN cohort and 72% of C3 Controls (*p* = 0.4) with a majority of C3GN patients receiving steroids alone (Table 3). Of the C3 control patients requiring immunosuppression, 40% were treated with two agents. Throughout the duration of follow up 70% of C3GN patients and 85% of C3 Control patients received rennin-angiotensin system (RAS) blockade.

**Table 3:** Distribution of outcomes and progression of diseases between both groups

Outcomes	C3GN (n = 10)	C3 (n = 21)	***P*** value
Follow up (days)	405 (203–1197)	1822 (1243–3948)	0.02
Remission with immunosuppression (%)	20 (n = 2)	33(n = 7)	0.5
Partial remission with immunosuppression (%)	10 (n = 1)	10 (n = 2)	0.9
Remission without immunosuppression (%)	Nil	19 (n = 4)	
Partial remission without immunosuppression (%)	Nil	Nil	
Relapse (%)	nil	5 (n = 1)	0.5
Creatinine doubling (%)	10 (n = 1)	19 (n = 4)	0.2
Median Creatinine doubling time (days)	350 NA	1107.5 (522–2076)	0.3
Median Creatinine doubling amount (umol/L)	353** NA	245 (229–277)	0.3
Latest Creatinine (umol/L)	125 (110–194)	110 (88–197)	0.8
Pre-emptive transplant (%)	0	14 (n = 3)	0.08
ESRF (HD/PD)	10 (n = 1)	14 (n = 3)	0.09
Death (% at 5 year follow up) ^a^	30 (n = 3)	14 (n = 3)	0.02
Loss to follow up (%)	30 (n = 3)	10 (n = 2)	0.1

Mann Whitney and Fishers exact test used. Logrank analysis used for ESRF and survival analysis. ^a^ Note not median single patient

Rates of complete remission in the C3GN group was 20% vs 33% in C3 Controls (*p* = 0.5), partial remission was 10% in both groups (*p* = 0.9), and relapse occurred in 5% of C3 Controls only (p = 0.5) (Table 3). The 20% of C3GN patients who ended up in remission received both steroids and MMF for the duration of follow up and did not relapse. Primary progressive disease was seen in one patient in both groups, whom did not receive any form of immunosuppression and progressed to ESRF (Tables 3 and 4). Overall 40% of C3GN patients did not receive immunosuppression versus 28% of C3 Controls (*p* = 0.7). Of C3GN patients who were not immunosuppressed, none achieved remission compared with 19% of C3 Controls (*p* = 0.04). Median duration of immunosuppression was 263 (IQR 126–629) and 302 (IQR 184–1496) days (p = 0.5) respectively. A smaller portion of C3GN patients received corticosteroids 30% vs 72% (*p* = 0.05) (Table 4). Those that did not progress to transplantation, ESRF or death show current median creatinine levels of 125 umol/L (IQR 88–197) and 110umol/L (IQR 110–194) and (*p* = 0.8) respectively (Table 3).

**Table 4:** Distribution of treatments received and progression of dieses between both groups

Treatment	C3GN (n = 10)	C3 Controls (***n*** = 21)	***p*** value
^**a**^**RAS blockers (%)**	70 (n = 7)	85 (n = 18)	0.3
**Steroids (%)**	30 (*n* = 3)	72 (*n* = 15)	0.05
^**b**^**MMF (%)**	20 (n = 2)	33 (n = 7)	0.4
^**c**^**Tac (%)**	0	10 (n = 2)	1
^**d**^**CP (%)**	10 (n = 1)	14 (n = 3)	1
^e^**CsA (%)**	0	19 (n = 4)	0.3
^f^**AZA (%)**	0	10 (n = 2)	1
**No immunosuppression (%)**	40 (n = 4)	28 (n = 6)	0.7
**Missing data (%)**	20 (n = 2)	0	0.09
**Immunosuppression (%)**	40 (n = 4)	72 (n = 15)	0.4
**Median Duration of immunosuppression (days)**	263 (IQR 126–629)	302 (IQR 184–1496)	0.5

Mann Whitney and Fishers exact test used

^**a**^*RAS* Renin-angiotensin system blockade, ^**b**^*MMF* Mycophenolate mofetil, ^**c**^*Tac* Tacrolimus, ^**d**^*CP* cyclophosphamide, ^e^*CsA* Ciclosporin, ^f^*AZA* azathioprine

### Progression to end-stage renal failure and survival

Progression to ESRF occurred in 14% of C3 Control patients and 10% of C3GN patients (log rank *p* = 0.08) over 5-year follow up (Table 3). No patients from the C3GN cohort underwent transplantation, whilst 14% of C3 Control patients received pre-emptive renal transplants (p = 0.08). Overall survival rates were lower in the C3GN group as compared to Controls (30% vs 14%), (log rank *p* = 0.02) (Table 3). However, mortality directly related to renal disease was seen in only one of three deceased patients in the C3GN group, whilst all patients in the C3 Control group suffered deaths related to renal complications.

## Discussion

Defining the histopathological and clinical differences between C3GN and other C3 predominating GNs remains challenging. Our review of 31 C3 predominant biopsy samples led to the reclassification of 10 of 31 patients as having C3GN. Our data highlights that clinical and serological features alone are not sufficient to distinguish between C3GN and other C3 predominant diseases. It does however show reclassification of these patients has an impact on diagnosis and outcomes.

Although our sample size is small, Caucasians were the most prevalent race in the C3GN group with a possible preponderance of disease in Torres Strait Islander patients. However, no underlying genetic mutation has been identified in this group [7, 9]. C3GN patients had a higher creatinine at diagnosis with 30% suffering an AKI requiring HD at onset. Pre-emptive transplantation occurred in 14% of the C3 Control only, without graft loss during the 5-year follow up period. A recent case series has demonstrated high rates of diseases reoccurrence in patients transplanted with C3G, despite maintenance immunosuppression and a variable response to eculizumab therapy [13]. In our cohort transplanted C3 Control patients maintained stable graft function without disease recurrence, suggesting these patients were classified into the correct underlying disease. During the follow up period no C3GN patient underwent transplantation, therefore direct assessment of disease reoccurrence between the two groups was not possible.

Fewer C3GN patients received immunosuppression (40% vs 72% in C3 Controls *p* = 0.4), the rate of remission was 20% vs 33% (*p* = 0.5) respectively. However, missing treatment data in 3 patients from the C3GN cohort, along with a total of 3 patients having early loss to follow up from both groups, must be taken into consideration given the small sample size.

Avasare et al., 2018, performed a retrospective chart analysis of 30 patients with C3GN, highlighting those treated with combination therapy of MMF and corticosteroids having higher rates of remission. They suggested improved prognosis in patients with lower proteinuria prior to initiation of treatment [17]. However, our C3GN patients had higher baseline proteinuria compared to C3 Controls, and 30% of the C3GN group also had nephrotic range proteinuria at diagnosis. Only 30% of C3GN patients had full or partial remission amongst those who received immunosuppression, and no remission was seen in patients without therapy. Our lower rates of remission may be attributed in part to the limited number of C3GN patients whom where administered immunosuppression (40%). Furthermore, treatment administered was based upon the original diagnosis at the time of biopsy, medication regimens were therefore targeted at the original diagnosis and may have been suboptimal therapy for C3GN.

Overall survival was lower in this C3GN cohort compared to Controls (logrank *p* = 0.02), however death related to renal disease or its complications was found in one of three patients only. Whilst the remaining two patients died either of solid organ malignancy or overwhelming sepsis. Therefore, it is unclear to what extent C3GN has directly impacted mortality in these patients. A recent paper published in the United states also showed poor prognosis and high rates of progression in both C3GN and DDD patients with combined primary outcome showing (39.1%) of C3GN and (41.7%) DDD patients had either doubling of serum creatinine, CKD stage 4–5, ESRF, death or transplantation [7]. However, there were no deaths amongst the C3GN cohort in that study [7]. Unlike other cohorts from France and Minnesota, where C3GN was related to a better prognosis compared to other C3G, our study has shown greater mortality C3GN group compared to the C3 Control group [8, 9]. Prognosis would also depend on C3GN related diseases, and at the time of diagnosis no patients in the C3GN cohort had no underlying haematological malignancies or autoantibody detected that may impact survival (Table 1). However, during the follow up of these patients repeat haematological and autoantibody screening was not undertaken to assess for delayed presentation of diseases.

In terms of histological assessment amongst the two groups, key differences were seen in EM findings. Notably, the C3GN patients all had EM deposits, as compared to 38% of Controls where deposits were not present (*p* = 0.02). These deposits were not electron dense in character, but rather granular formless deposits in the mesangium, subepithelium and subendothelium (Fig. 2). Similar to other studies [6–8], our results indicate the nature and location of EM deposits is potentially critical in the differentiation of C3GN from other C3 predominant diseases. In terms of disease activity, there was no difference in crescent, or leukocyte infiltration, however C3GN patients had a greater endocapillary proliferation 40% vs 14% (*p* = 0.2).

**Fig. 4:**
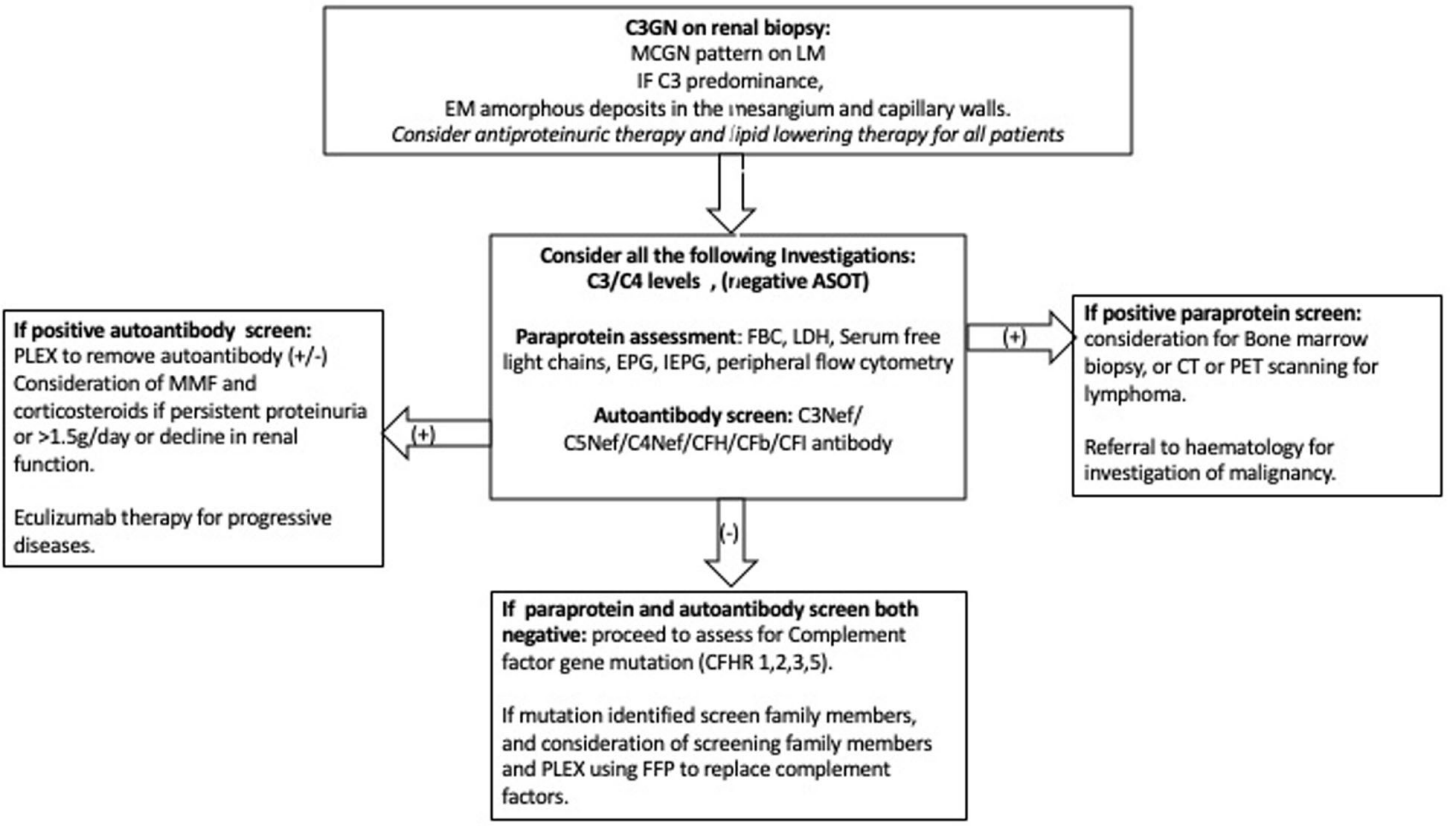
Diagnosis and treatment algorithm for C3GN used at The Canberra Hospital

This is first cohort of Australian patients with reclassified C3GN, we note a higher a than expected portion Torres Strait islander patients in the C3GN cohort. Based on our retrospective analysis and literature review, we have devised an investigation and treatment algorithm used in our institution to systematically diagnose and treat C3GN (Fig. 4). This algorithm utilizes biopsy characteristics [7, 9], along with assessment for malignancy, autoantibodies and possibly genetic sequencing to assist in discovering the aetiology of disease in patients. Haematology referral, immunosuppression and possible plasmapheresis are considered viable options once the aetiology of C3GN is uncovered, whilst all patients should be considered for antiproteinuric agents and lipid lowering therapy [1, 13, 18, 19] (Fig. 4).

Limitations of our study include sample size and its retrospective nature. Due to the recent characterization of C3GN, more prospective samples are required to characterise this disease and assess clinical remission, however, there remains a risk for ascertainment bias with emergent diagnoses. Given the limited number of samples meeting C3GN criteria, drop-out rates also significantly impacted the assessment of long term out comes and disease progression.

## Conclusion

Clinical and serological features alone are not sufficient to distinguish C3GN from other C3 predominant glomerulopathies. In this cohort C3GN patients presented with a greater degree of renal injury from disease onset and higher mortality rate. Histological assessment revealed C3GN patients are likely to have to have IF pattern with C3 staining alone. We observed the same pattern of deposition on EM as described in other series of C3GN patients. Our study, highlights C3GN may have a racial predisposition in ethnic minorities. C3GN has poor outcomes, and in our cohort, we note remission without immunosuppression is unlikely, however the impact of treatment itself shows limited efficacy. Larger prospective studies are required to better characterise this disease and treatment efficacy in the Australian population.

## Data Availability

The datasets used and/or analysed during the current study are available from the corresponding author on reasonable request.

## Abbreviations

3GN: C3 glomerulonephritis
MPGN: Membranoproliferative glomerulonephritis
IC: Immune complex-mediated disease
ASOT: Anti-streptolysin titres
GBM: Glomerular basement membrane
DDD: Dense deposit diseases
ESRF: End-stage renal failure
PIGN: Post-infectious GN
C3G: C3 glomerulopathy
IF: Immunofluorescence
ACT: Australian Capital territory’s
upcr: Protein/creatinine ratio
KDIGO: Kidney Diseases Improving Global Outcomes
LN: Lupus nephritis
AKI: Acute Kidney injury
HD: Haemodialysis
LM: Light microscopy
EM: Electron microscopy
RAS: Rennin-angiotensin system
MMF: Mycophenolate mofetil
Tac: Tacrolimus
CP: Cyclophosphamide
CsA: Ciclosporin
AZA: Azathioprine
